# Flexible supplier selection and order allocation in the big data era with various quantity discounts

**DOI:** 10.1371/journal.pone.0283585

**Published:** 2023-03-27

**Authors:** Qing Wang

**Affiliations:** Department of Intelligence Science and Technology, Shanghai Lixin University of Accounting and Finance, Shanghai, China; Bond University, AUSTRALIA

## Abstract

This paper studies the flexible large-scale supplier selection and order allocation problem with various quantity discounts, i.e., no discount, all-unit discount, incremental discount, and carload discount. It fills a literature gap that models usually formulate one or seldom two types because of the modeling and solution difficulty. All suppliers offering the same discount are far from reality, especially when the number of suppliers is large. The proposed model is a variant of the NP-hard knapsack problem. The greedy algorithm, which solves the fractional knapsack problem optimally, is applied to cope with the challenge. Three greedy algorithms are developed using a problem property and two sorted lists. Simulations show the average optimality gaps are 0.1026%, 0.0547%, and 0.0234% and the model can be solved in centiseconds, densiseconds, and seconds for supplier numbers 1000, 10000, and 100000. This allows the full use of data in the big data era.

## Introduction

For decades, supplier selection has been an extensive research topic [1–3]. Often, it combines with order allocation if one supplier cannot supply all the quantities [4, 5]. It associates with quantity discounts when suppliers offer discount schemes. Today’s markets are changing, and customer tastes vary and are difficult to predict. Manufacturers should meet unique needs of color, shape, material, decoration, and others. Flexibility in choosing suppliers is necessary to meet specific requirements. Quick decisions are desired to save time, speed up order response, and improve the user experience.

In the era of big data, massive amounts of data are collected, stored, transferred, and processed [6–8]. Leveraging big data helps companies to improve the quality and agility of decision-making and brings great business value. With big data, suppliers can be selected from a much larger pool. More alternatives are provided to the decision-makers, which results in higher profits or lower costs. Large-scale models are developed. For NP-hard models, such as knapsack problems, conventional algorithms suffer from the "curse of dimensionality". They take long solution times or are unable to solve within a reasonable time limit–one or several hours.

The fractional knapsack problem formulates the original supplier selection and order allocation problem. The greedy algorithm is efficient and solves it to optimality. The knapsack formulation becomes more complex when setup costs and various quantity discounts are involved. Whether the greedy algorithm can still provide good solutions becomes questionable.

This paper studies flexible supplier selection and order allocation in the big data era with setup and four quantity discount types. A two-layer framework makes use of big data and screens technical requirements. The proposed nonlinear integer programming model is a variant of the knapsack problem. Three greedy algorithms are progressively developed by leveraging an underlying mechanism and utilizing two sorted lists. The lists use actual unit costs to balance the setup costs and different quantity discounts. The main contributions of this paper are twofold:

It proposes a nonlinear integer programming model that formulates four common types of quantity discounts. It fills a gap in the literature where models consider a limited number of discount types. These models are not suitable for practical applications.

The exact algorithms encounter the “curse of dimensionality” for solving the proposed model. To fully exploit the big data, this paper develops three greedy algorithms capable of solving very large-scale problems (up to 100000 suppliers) in seconds, with average optimality gaps of 0.1026%, 0.0547%, and 0.0234%.

The remaining paper is organized as follows. Section 2 reviews the relevant literature. Section 3 gives the formulation of the model. Section 4 presents the greedy algorithms for solving the large-scale model. A numerical study is provided in section 5, and computational experiments are conducted in section 6. Section 7 concludes the paper.

## Literature review

### Flexible supplier selection

Flexible decisions are desired in supply chains to increase resilience and agility. [9] evaluated supply, manufacturing, and logistics flexibility with the developed stochastic programming model. [10] developed a flexible dynamic sustainable procurement model to cope with the uncertainties of global supply chains. [11] modeled flexible sustainable supplier selection decisions in multitier supply chains.

### Supplier selection with quantity discount

A literature study on supplier selection with quantity discounts shows that most models have one type of quantity discount [12–14]. A few have two [15, 16]. It is not realistic, especially when the number of suppliers is large.

Different quantity discount schemes bear different cost structures. The no discount cost is linear, the incremental discount cost is concave, the all-units discount cost is discontinuous, and the carload discount cost is a convex polyline [17, 18]. Models formed with one or two types often cannot describe those with more. Supplier selection models are NP-hard [19]. As the number of discounts increases, modeling complexity and solution difficulty increase. Models with various discounts and their efficient solutions are demanded in the era of big data.

### Knapsack problem

Knapsack problems are extensively studied combinatorial optimization problems with a wide range of applications [20, 21] and many variants of forms. Knapsack problems are NP-hard. [22, 23] reviewed variants of the knapsack problems and solution algorithms and heuristics, structured in two papers. 0–1 knapsack is the most traditional form. In the 0–1 knapsack, objects cannot be partitioned, i.e., either selected or not selected. In the fractional knapsack, part of an object can be selected. The greedy algorithm chooses greedily the part with the highest value, which can lead to an optimal solution [21]. Though it can solve the fractional knapsack problem perfectly, it is not an optimal algorithm for other knapsack problems.

### Curse of dimensionality

When applying exact solution algorithms like dynamic programming or branch-and-bound to solve knapsack or other combinatorial optimization problems, the “curse of dimensionality” will occur. The “curse of dimensionality”, introduced by Bellman for dynamic programming, indicates the exponential growth of hypervolume as a function of dimensionality [24, 25]. It leads to an exponential growth of the solution time as the dimensions of the problem increase. For large problem sizes, it may not be possible to solve within a reasonable time limit (one or several hours).

[26] proposed a branch-and-bound algorithm for solving an integer quadratic multi-knapsack problem. Table 1 in the paper shows that when the problem dimensions *m* and *n* are increased from 100 to 2000, the solution time of the four exact algorithms increases rapidly from less than/greater than one second to more than the time limit of 3 hours. [27] developed a Lagrangian-based branch-and-cut algorithm for the interval min-max regret generalized assignment problem. Table 3 in the paper shows that when the dimension *m* increases to 10 and *n* increases to 80, the problem soon becomes unsolvable by the exact algorithms—Benders-like decomposition, basic branch-and-cut, and Lagrangian-based branch-and-cut, within a time limit of 3600 seconds. [28] proposed an exact branch-and-bound algorithm for the quadratic combinatorial optimization problem. Table 4 in the paper shows the very fast increase in the computation time for three solvers and two algorithms, from a few seconds to several hours or over the time limit, when the number of variables increases from 180 to 420.

**Table 1:** Indices.

Indices	Meaning
*i*	Index of suppliers, *i* = 1,…,*n*
*j*	Index of discount intervals, *j* =1,…,*M*_*i*_

### Greedy algorithm

The greedy algorithm is a solution scheme that greedily makes the best choice at the current stage. It can solve the fractional knapsack problem to optimality. Greedy algorithms obtain solutions efficiently, but the quality doesn’t guarantee. [29] proposed greedy algorithms to solve two-dimensional knapsack problems with binary weights. [30] developed greedy algorithms for linear and quadratic knapsack problems. Other than knapsack problems, greedy algorithms apply in many areas. [31] presented a greedy algorithm for the maximization of sequence submodular functions. [32] developed a greedy policy for the dynamic information provision model. [33] applied greedy algorithms to solve the single-demand facility location problem.

### Flexible supplier selection and order allocation in the big data era with various quantity discounts

**Fig 1:**
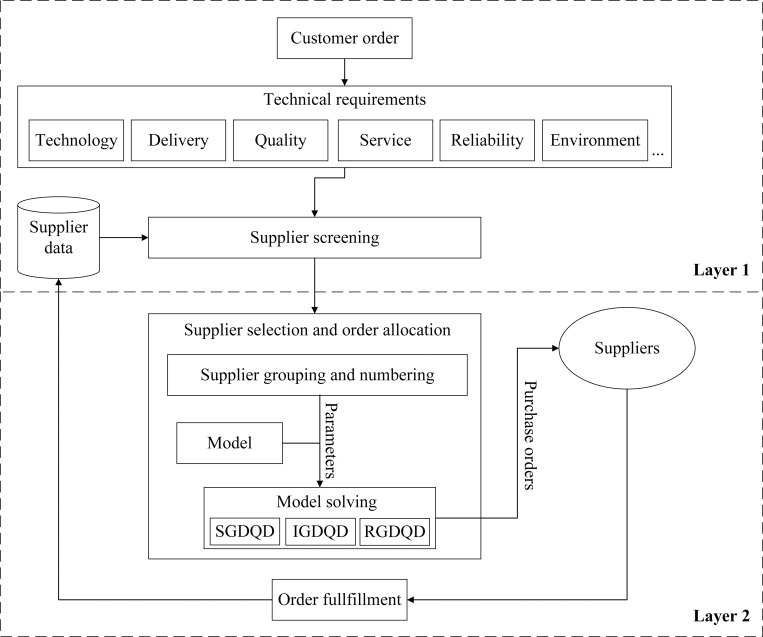
Two-layer framework.

The two-layer framework for flexible supplier selection and order allocation in the big data era is shown in Fig 1.

### Layer 1: Supplier screening

When a customer order arrives, its technical requirements are identified. These requirements may include specific technologies, delivery lead times, delivery reliability, quality standards, services to provide, materials to use, the durability of the product, sustainability issues, etc. The requirements are converted into codes to match the data stored in the database. For instance, technology is stored in the database by its code *T*_*k*_. The order requires technologies *T*_3_ and *T*_10_. Suppliers must possess the technologies to fulfill the order. Data like the percentage of late delivery and average lead time are recorded and updated after the order fulfillment. In case of urgent orders, suppliers with short average lead times satisfy. Suppliers are screened to obtain a list of suppliers that meet all the requirements.

### Layer 2: Supplier selection and order allocation

The list of suppliers is grouped by the pricing schemes and numbered. The supplier data form the model parameters, for instance, *S*_*i*_, *c*_*ij*_. Combining them with the model stored in the system’s model library produces the mathematical programming model to be solved. A nonlinear integer programming model is proposed in this paper. Three algorithms, SGDQG, IGDQD, and RGDQD, are proposed for solving the model efficiently. The proposed model and the algorithms are elaborated below.

After order fulfillment, the performance data are used to update the database.

### Model formulation

**Fig 2:**
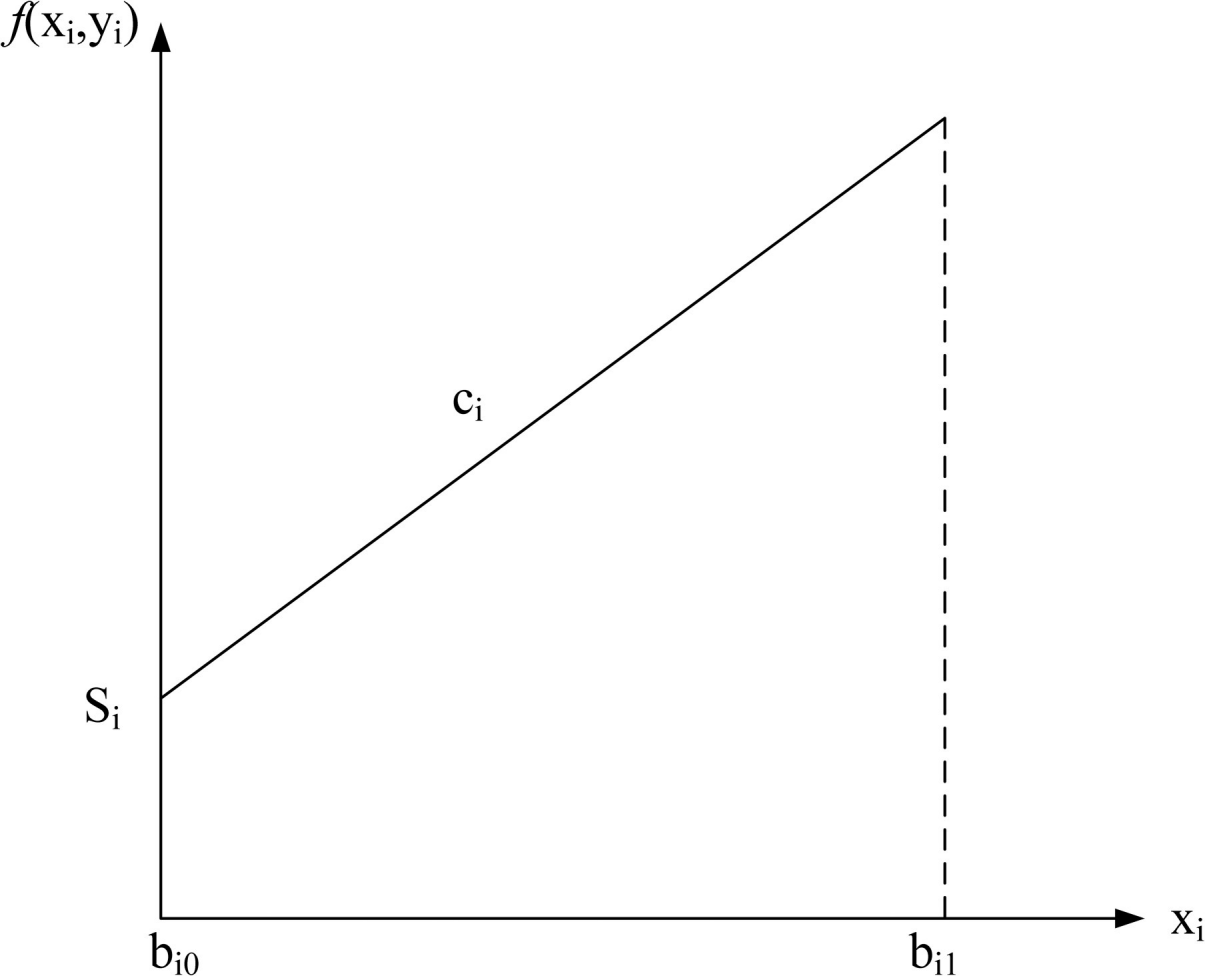
No discount.

**Fig 3:**
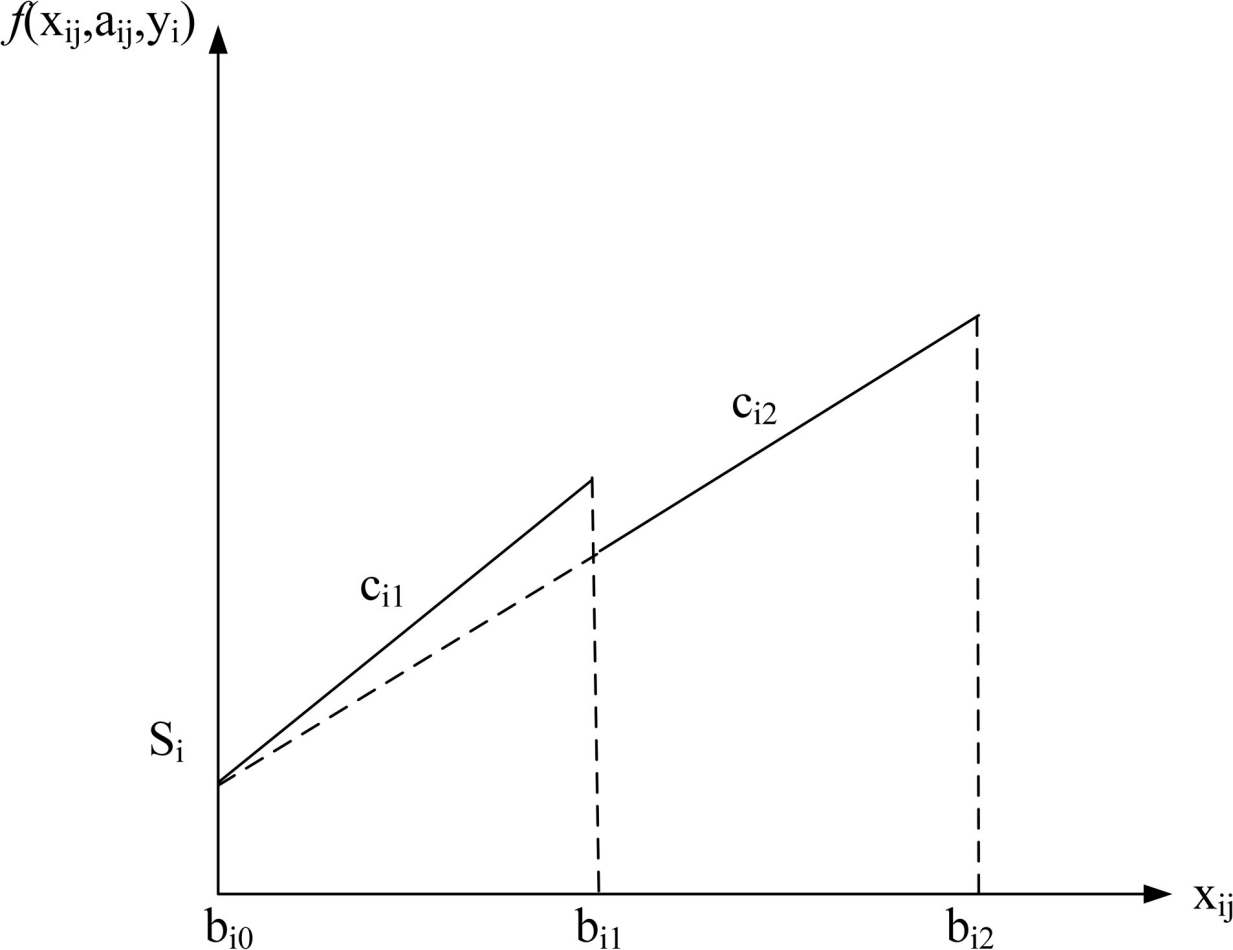
All-units discount (2 intervals).

**Fig 4:**
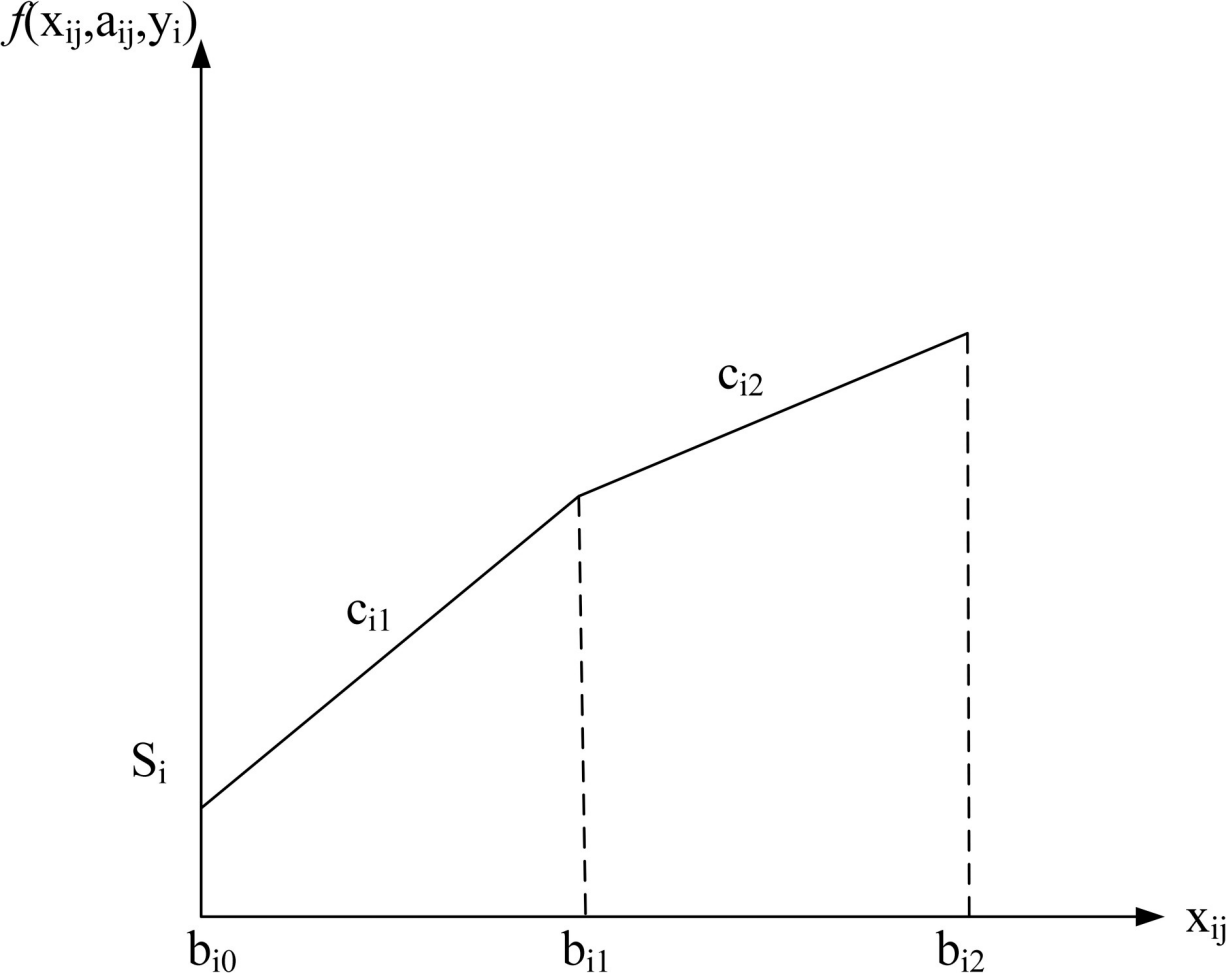
Incremental discount (2 intervals).

**Fig 5:**
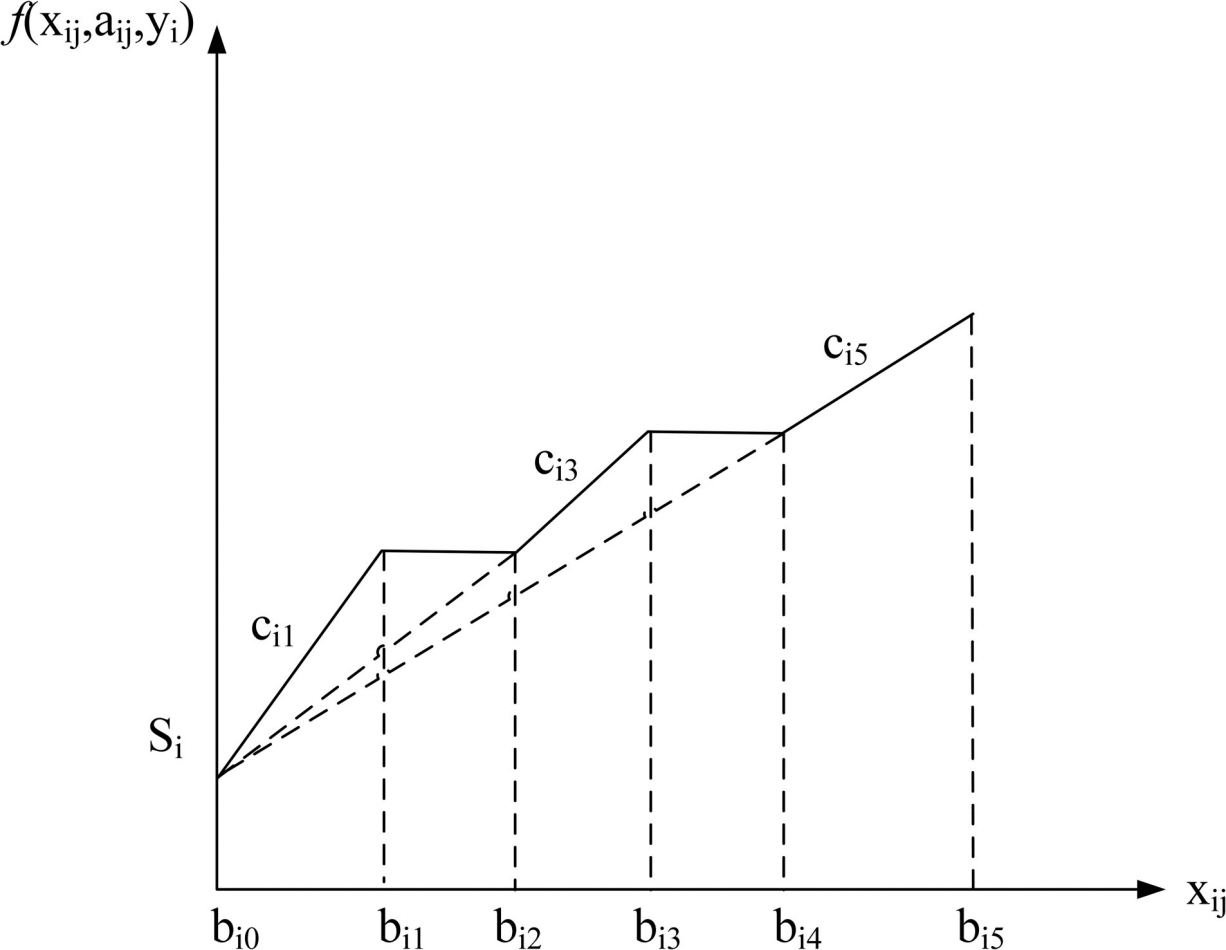
Carload discount (5 intervals).

Four common pricing schemes are formulated, namely no discount, all-units discount, incremental discount, and carload discount. The cost structure for each discount type is shown in Figs 2–5. The horizontal axis of the figures denotes the order quantity and the vertical axis denotes the cost. The indices, parameters, and decision variables used in the figures and models are listed in Tables 1–3. First, the model for each type is formulated separately.

**Table 2:** Parameters.

Parameters	Meaning
*S*_*i*_	Setup cost of supplier *i*, including ordering and transportation costs, $
*c*_*i*_	The unit cost of supplier *i* for the no discount case, $
*Q*	Total order quantity, units
*b*_*ij*_	Boundary value for interval *j* of supplier *i*, *b*_*i*0_ = 0, only 1 interval for the no discount case, units
*c*_*ij*_	Unit cost for interval *j* of supplier *i*, $
*M*_*i*_	Number of discount intervals of supplier *i*, integers
*K*_*ij*_	Aggregated purchase cost till the jth interval for supplier *i* in the incremental discount case, calculated as $K_{ij}=K_{i,j-1}+c_{ij}(b_{ij}-b_{i,j-1}),K_{i0}=0$, $

**Table 3:** Decision variables.

Decision variables	Meaning
*y*_*i*_	Binary variable denoting whether supplier *i* is selected
*x*_*i*_	Order quantity of supplier *i* in the no discount case, integers
*x*_*ij*_	Order quantity of supplier *i* in interval *j*, integers
*a*_*ij*_	Binary variable denoting whether interval *j* of supplier *i* is selected

**No discount case.** The model for the no-discount case is formulated as follows.


$$\min\sum_{i=1}^{n}{(y}_{i}S_{i}+c_{i}x_{i})$$ (1)

$$s.t.\sum_{i=1}^{n}x_{i}=Q,$$ (2)

$$y_{i}\leq x_{i}\leq b_{i1}y_{i},i=1,\ldots ,n,$$ (3)

$$x_{i}\;integers,i=1,\ldots ,n,$$ (4)

$$y_{i}\in \{0,1\},\forall i=1,\ldots ,n.$$ (5)

The objective function (1) minimizes the total cost, which is composed of the setup cost and the unit cost. Constraint (2) ensures that the total order quantity is equal to *Q*. Constraint (3) restricts the order quantity *x*_*i*_ of supplier *i* to be between 0 and the supplier capacity *b*_*i*1_ if supplier *i* is selected, or 0 if supplier *i* is not selected. Constraints (4) and (5) ensure that decision variables *x*_*i*_ and *y*_*i*_ are integer and binary variables, respectively.

**All-units discount case.** The model for the all-units discount case is as follows.


$$\min\sum_{i=1}^{n}{(y}_{i}S_{i}+\sum_{j=1}^{M_{i}}a_{ij}c_{ij}x_{ij})$$ (6)

$$s.t.\sum_{i=1}^{n}\sum_{j=1}^{M_{i}}x_{ij}=Q,$$ (7)

$$a_{ij}b_{i,j-1}\leq x_{ij}\leq a_{ij}b_{ij},i=1,\ldots ,n,j=1,\ldots ,M_{i},$$ (8)

$$\sum_{j=1}^{M_{i}}a_{ij}=y_{i},i=1,\ldots ,n,$$ (9)

$$a_{ij}\in \{0,1\},i=1,\ldots ,n,j=1,\ldots ,M_{i},$$ (10)

$$y_{i}\in \{0,1\},i=1,\ldots ,n,$$ (11)

$$x_{ij}\;integers,i=1,\ldots ,n,j=1,\ldots ,M_{i}.$$ (12)

The objective function (6) minimizes the summation of the setup costs and the unit costs. Constraint (7) equals the summation of quantities *x*_*ij*_ to the total order quantity *Q*. Constraint (8) denotes if interval *j* for supplier *i* is selected, i.e., *a*_*ij*_ =1, the purchase amount *x*_*ij*_ must be between the interval boundaries, otherwise, *x*_*ij*_ = 0. Constraint (9) denotes if supplier *i* is selected, one and only one discount interval for the supplier must be selected. Constraint (10)-(12) restrict *a*_*ij*_, *y*_*i*_ and *x*_*ij*_ to be integer and binary variables.

**Incremental discount case.** The model for the incremental discount case is as follows.


$$\min\sum_{i=1}^{n}{\{y}_{i}S_{i}+\sum_{j=1}^{M_{i}}a_{ij}[{K_{i,j-1}+c}_{ij}(x_{ij}-b_{i,j-1})]\}$$ (13)

The objective function (13) minimizes the summation of the setup costs and the unit costs for the incremental discount. The constraints are the same as in the all-units discount case.

**Carload discount case.** The model for the carload discount case is as follows.


$$\min\sum_{i=1}^{n}{[y}_{i}S_{i}+\sum_{j=1}^{(M_{i}-1)/2}\left(a_{i,2*j-1}c_{i,2*j-1}x_{i,2*j-1}+{{a_{i,2*j}c}_{i,2*j-1}b}_{i,2*j-1})+a_{iM_{i}}c_{iM_{i}}x_{iM_{i}}\right]$$ (14)

The objective function (14) minimizes the summation of the setup costs and the unit costs for the carload discount case, the odd and even intervals have different cost structures as shown in Fig 5, and are calculated separately. The constraints are the same as in the all-units discount case.

### Supplier selection and order allocation model with setup and various quantity discounts

The above models are integrated into one model that incorporates all four types of pricing schemes for supplier selection.


$$(P)\;\min\sum_{i=1}^{n_{1}}{(y}_{i}S_{i}+c_{i}x_{i})+\sum_{i=n_{1}+1}^{n_{2}}{(y}_{i}S_{i}+\sum_{j=1}^{M_{i}}a_{ij}c_{ij}x_{ij})$$

$$+\sum_{i=n_{2}+1}^{n_{3}}{\{y}_{i}S_{i}+\sum_{j=1}^{M_{i}}a_{ij}[{K_{i,j-1}+c}_{ij}(x_{ij}-b_{i,j-1})]\}$$

$$+\sum_{i=n_{3}+1}^{N}{[y}_{i}S_{i}+\sum_{j=1}^{(M_{i}-1)/2}\left(a_{i,2*j-1}c_{i,2*j-1}x_{i,2*j-1}+{a_{i,2*j}c_{i,2*j-1}b}_{i,2*j-1})+a_{iM_{i}}c_{iM_{i}}x_{iM_{i}}\right]$$ (15)

$$s.t.\sum_{i=1}^{n_{1}}x_{i}+\sum_{i=n_{1}+1}^{N}\sum_{j=1}^{M_{i}}x_{ij}=Q,$$ (16)

$$y_{i}\leq x_{i}\leq b_{i1}y_{i},i=1,\ldots ,n_{1},$$ (17)

$$a_{ij}b_{i,j-1}\leq x_{ij}\leq a_{ij}b_{ij},i=n_{1}+1,\ldots ,N,j=1,\ldots ,M_{i},$$ (18)

$$\sum_{j=1}^{M_{i}}a_{ij}=y_{i},i=n_{1}+1,\ldots ,N,$$ (19)

$$a_{ij}\in \{0,1\},i=n_{1}+1,\ldots ,N,j=1,\ldots ,M_{i},$$ (20)

$$y_{i}\in \{0,1\},i=1,\ldots ,N,$$ (21)

$$x_{i}\;integers,i=1,\ldots ,n_{1},$$ (22)

$$x_{ij}\;integers,i=n_{1}+1,\ldots ,N,j=1,\ldots ,M_{i}.$$ (23)

Problem (P) is a nonlinear integer programming model. It can be perceived as some combination of 0–1 knapsack, multiple choice knapsack, fractional knapsack, and knapsack with setup problems, and is thus NP-hard. Problem (P) has $N+n_{1}+2\sum_{i=n_{1}+1}^{N}M_{i}$ decision variables and $2N+n_{1}+3\sum_{i=n_{1}+1}^{N}M_{i}+1$ constraints.

### Solution methodology

Solution methodologies are sought for to solve problem (P) efficiently. The following proposition shows a property of the solution of (P).

**Proposition 1:** There exists an optimal solution to problem (P) in which at most one quantity value *x*_*i*_ or *x*_*ij*_ lies strictly between two breakpoints.

**Proof:** Denote *x*_*i*_ or *x*_*ij*_ by *q*_*i*_, suppose there are two suppliers *d* and *e* that get non-breakpoint orders, $b_{dk}<q_{d}<b_{d,k+1}$ and $b_{el}<q_{e}<b_{e,l+1}$. Suppose *c*_*d*_≤*c*_*e*_. Then switching a partial order $\delta =min\{b_{d,k+1}-q_{d},q_{e}-b_{el}\}$ from supplier *e* to supplier *d* can reduce the total cost by at least (*c*_*e*_−*c*_*d*_)*δ*. After switching, either supplier *d* or supplier *e* get breakpoint order.

With Proposition 1, since the amount *Q* is an integer, and all boundary values are integers, the only non-boundary value must be an integer. The integer constraints of *x*_*i*_’s and *x*_*ij*_’s, i.e. (22) and (23), can be relaxed. From Proposition 1, the complexity of (P) may be reduced. A direct way of solving (P) is to iterate through all fractional intervals and compare the objective values. Let *f* denote the index of the supplier with the non-boundary interval. Problem (P) can be transformed into problem (P*f*).

$$(Pf)\;\min\sum_{i=1}^{n_{1}}{(y}_{i}S_{i}+c_{i}x_{i})+\sum_{i=n_{1}+1}^{n_{2}}{(y}_{i}S_{i}+\sum_{j=1}^{M_{i}}a_{ij}c_{ij}^{\prime }x_{ij})$$

$$+\sum_{i=n_{2}+1}^{n_{3}}{\{y}_{i}S_{i}+\sum_{j=1}^{M_{i}}a_{ij}[{K_{i,j-1}+c}_{ij}(x_{ij}-b_{i,j-1})]\}$$

$$+\sum_{i=n_{3}+1}^{N}{[y}_{i}S_{i}+\sum_{j=1}^{(M_{i}-1)/2}\left(a_{i,2*j-1}c_{i,2*j-1}x_{i,2*j-1}+{a_{i,2*j}c_{i,2*j-1}b}_{i,2*j-1})+a_{iM_{i}}c_{iM_{i}}x_{iM_{i}}\right]$$ (24)

$$s.t.\sum_{i=1}^{n_{1}}x_{i}+\sum_{i=n_{1}+1}^{N}\sum_{j=1}^{M_{i}}x_{ij}=Q,$$ (25)

$$\sum_{j=1}^{M_{i}}a_{ij}=y_{i},i=n_{1}+1,\ldots ,N,$$ (26)

$$b_{f,j-1}\leq f\leq x_{f}b_{fj},$$ (27)

$$x_{i}=b_{i1}y_{i},i=1,\ldots ,n_{1}\;and\;i\neq f,$$ (28)

$$x_{ij}=a_{ij}b_{ij},i=n_{1}+1,\ldots ,N\;and\;i\neq f,j=1,\ldots ,M_{i},$$ (29)

$$a_{ij}\in \{0,1\},i=n_{1}+1,\ldots ,N\;and\;i\neq f,j=1,\ldots ,M_{i},$$ (30)

$$y_{i}\in \{0,1\},i=1,\ldots ,N\;and\;i\neq f,$$ (31)

$$a_{f}=1,$$ (32)

$$y_{f}=1.$$ (33)

*c*_*ij*_ in objective function (15) is changed to $c_{ij}^{\prime }$ such that $c_{ij}^{\prime }=c_{i,j+1},\forall j<M_{i}$ and $c_{iM_{i}}^{\prime }=c_{iM_{i}}$. By substituting the equality constraints (28), (29), (32) and (33) into the objective function (24), the problem sizes of (P*f*) are reduced to $N+\sum_{i=n_{1}+1}^{N}M_{i},1\leq f\leq n_{1}$ or $N+1+\sum_{i=n_{1}+1,i\neq f}^{N}M_{i},n_{1}+1\leq f\leq N$ decision variables and $2N-n_{1}+\sum_{i=n_{1}+1}^{N}M_{i}+1,1\leq f\leq n_{1}$ or $2N-n_{1}+\sum_{i=n_{1}+1,i\neq f}^{N}M_{i}+1,n_{1}+1\leq f\leq N$ constraints, but the problem needs to be solved $n_{1}+\sum_{i=n_{1}+1}^{N}M_{i}$ times as *f* iterates through the intervals.

Though problem sizes reduce from (P) to (P*f*), (P*f*) is a variant of the knapsack problem, and is thus NP-hard. The complexity needs to be further reduced.

## Large-scale problem

In the big data era, great vendor information may be utilized, and large-scale problem (P) needs to be solved. However, exact solution algorithms suffer from the “curse of dimensionality”. This section seeks efficient solution methodologies for the large-scale problem (P).

As in the “Literature review” section, the greedy algorithm solves the fractional knapsack problem to optimality and is applied to solve other knapsack problems. Whether it can be a good algorithm for (P) will be investigated. To design a greedy algorithm for (P), how to make the greedy choice is the key issue. The fractional knapsack greedily selects the unit revenue. (P) aims to find the lowest total cost, dividing by the order quantity *Q*, which is the same as finding the lowest average cost. Filling the knapsack greedily with the lowest actual cost per unit accounting for all the costs becomes a good choice.

For the fractional knapsack, the unit revenues can be calculated and sorted. The unit revenue doesn’t change for a single object, no matter how many quantities of the object are selected. This makes the greedy selection possible. But with setup costs and different discounts, the actual cost per unit varies with quantity, making the sorting tedious.

Proposition 1 shows that in an optimal solution to (P), all but one of the quantity values are boundary values. The actual unit costs of boundary values can be calculated and sorted. Greedy choices can be made based on these costs. This forms List 1 below. The ordering of actual unit costs for the remaining quantity forms List 2. The proposed greedy algorithms in the following are based on these two sorted lists.

### Greedy algorithms

The actual unit cost is defined as the average unit cost of ordering a single item, considering all the costs incurred. The calculation for a particular *x*_*i*_ or *x*_*ij*_ differs for different quantity discount types, and is given in Eqs (34)–(38).

For no discount,

$${ac}_{i}=c_{i}+\frac{S_{i}}{x_{i}}.$$ (34)

For all-units discount,

$${ac}_{ij}=c_{ij}+\frac{S_{i}}{x_{ij}}.$$ (35)

For incremental discount,

$${ac}_{ij}=\frac{K_{i,j-1}+c_{ij}(x_{ij}-b_{i,j-1}){+S}_{i}}{x_{ij}}.$$ (36)

For carload discount, for quantities between odd intervals,

$${ac}_{ij}=c_{ij}+\frac{S_{i}}{x_{ij}},$$ (37)

for quantities between even intervals,

$${ac}_{ij}=\frac{c_{ij}b_{ij}+S_{i}}{x_{ij}}.$$ (38)

List 1 and List 2 are obtained as follows.

List 1: Rank ascendingly the actual unit costs of boundary quantity *b*_*ij*_’s for all supplier *i* and interval *j*, and mark the respective supplier no. *i*, interval no. *j*, and boundary quantity *b*_*ij*_.

List 2: Rank ascendingly the actual unit costs of a certain quantity Δ*Q* for all supplier *i* and interval *j*, and mark the associated supplier no. *i*, interval no. *j*, where the suppliers no. is not selected in List 1. If the quantity is in the range of an interval *j*, i.e., *b*_*i*,*j*−1_<Δ*Q*<*b*_*ij*_, use the quantity Δ*Q* to calculate the actual unit cost for interval *j*; If the quantity exceeds the range of an interval *j*, i.e., *b*_*ij*_≤Δ*Q*, use the interval’s boundary value *b*_*ij*_ to calculate the actual unit cost for interval *j*; Otherwise if the quantity is below the range of interval *j*, i.e., *b*_*i*,*j*−1_≥Δ*Q*, the actual unit cost for interval *j* is assigned a large number, e.g., 1000, and interval *j* will not appear in the rank of List 2. Mark the associated quantity of each ranked item with the quantity to calculate the actual unit cost.

In List 2, interval *j*’s whose upper boundary *b*_*ij*_ less than Δ*Q* are also taken into account, since some combination of intervals up in the list may give better results.

First, the simple greedy algorithm is proposed. The procedure is as follows.

**Algorithm SGDQD:** *Simple greedy algorithm for supplier selection with different quantity discounts*

Step 1. Obtain List 1 with no repetitive supplier *i* until adding the next item will exceed *Q*. Obtain the sum of boundary quantity *b*_*ij*_’s in the list as *Q*′. Calculate Δ*Q* = *Q*−*Q*′.

Step 2. Obtain List 2 with one item for the quantity Δ*Q*. Let *q* be the associated quantity, calculate Δ*Q* = Δ*Q*−*q*.

Step 3. Repeat Step 2 until Δ*Q* = 0.

SGDQD is simple, and a spreadsheet can be used to find solutions. Place the supplier data in the cells of the spreadsheet and use "function" to get sorted lists—List 1 and List 2. Regardless of the size of the supplier set, SGDQD only needs to get the top “*n*” suppliers of List 1 and the topmost supplier of List 2. Solvers need to solve a mathematical programming problem with large dimensions, which is complex and time-consuming. SGDQD can attain the optimal solution for some problems, but improvements are possible. More calculations and comparisons are performed. The following improved greedy algorithm are obtained.

**Algorithm IGDQD:** *Improved greedy algorithm for supplier selection with different quantity discounts*

Step 1. Obtain List 1 with no repetitive supplier *i* until adding the next item will exceed *Q*. Obtain the sum of boundary quantity *b*_*ij*_’s in the list as *Q*′. Calculate Δ*Q* = *Q*−*Q*′.

Step 2. Obtain List 2 for the quantity Δ*Q* until the associated quantity of an item equals Δ*Q*. Form trajectories for the items in List 2. The last item is an end of trajectory. If a single item is obtained, the associated trajectory ends.

Step 3. For items except the last item, let q be the associated quantity, calculate Δ*Q* = Δ*Q*−*q*, and repeat Step 2.

Step 4. Calculate the total cost for each trajectory in the obtained tree and select the minimum. The associated trajectory gives the optimal solution.

**Fig 6:**
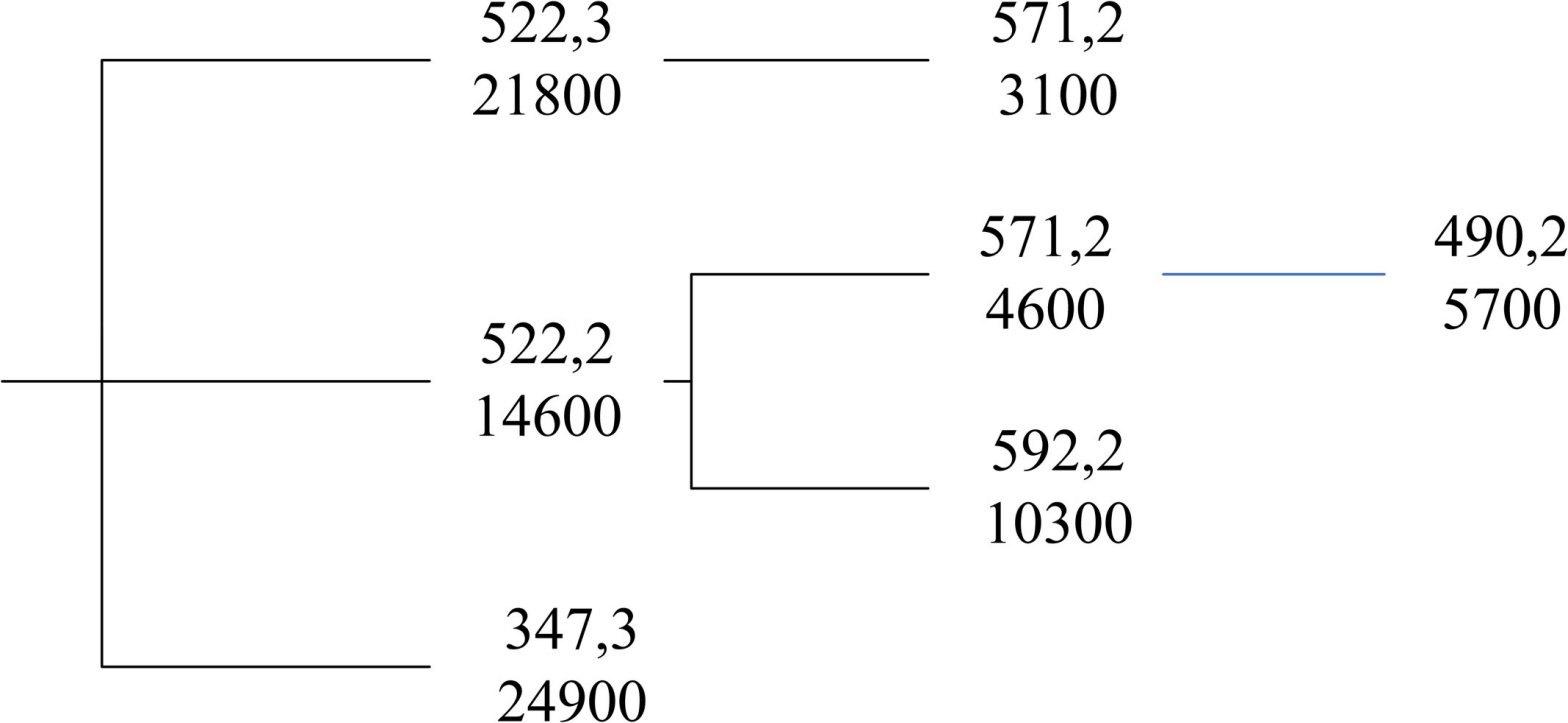
Trajectories formed, Data20.

Step 1 in IGDQD is the same as in SGDQD, i.e., List 1 is used the same to fill *Q*. More calculations are performed on List 2 to obtain the combination with the lowest cost for the quantity gap of Step 1. An example of the calculations is given using randomly generated Data20. Table 4 shows List 1 for *Q* = 300000 without allowing duplicate supplier numbers. The quantity gap of Step 1 is 24900. Then, as shown in Table 5, List 2 is obtained for the amount 24900. The trajectories are formed as shown in Fig 6. List 2’s for the amounts 3100, 10300, and 5700 are obtained to form the rest of the trajectories in Fig 6. In this case, the uppermost trajectory gives the minimum total cost.

**Table 4:** List 1 for *Q* = 300000, Data20, no repetitive supplier no. allowed.

Actual unit cost	Supplier no.	Boundary no.	Boundary quantity
9.242809278	516	4	38800
9.522621723	933	6	53400
9.528230384	901	5	59900
9.543367876	486	4	38600
9.596296296	372	2	18900
9.649225806	515	4	31000
9.682458101	915	7	17900
9.689216867	464	4	16600

**Table 5:** List 2 of quantity 24900, Data20.

Actual unit cost	Supplier no.	Boundary no.	Boundary quantity
9.878944954	522	3	21800
9.917876712	522	2	14600
9.974096386	347	3	24900

In rare cases, List 2 has many small quantities. Many replicated trajectories are present. To further simplify, combinations of the quantities are calculated and compared.

IGDQD improves the solutions to some extent by exploring List 2 more. In fact, List 1 can also be explored to obtain better solutions. The refined greedy algorithm improves IGDQD by performing calculations on both List 1 and List 2.

**Algorithm RGDQD:** *Refined greedy algorithm for supplier selection with different quantity discounts*

Step 1. Obtain List 1. Select and add boundary quantity *b*_*ij*_’s in List 1 by order with no repetitive supplier no. allowed until adding the next item will exceed *Q*. The last selected quantity has the rank no. *l* in List 1. Obtain the quantities selected whose rank no. is less than *l*−*d*_1_+1, enumerate and select the quantities in List 1 from the rank no. *l*−*d*_1_+1 to *l*+*d*_2_ with no repetitive supplier no. *i* such that *Q* is not exceeded. Obtain the sum of boundary quantity *b*_*ij*_’s for each combination of selected items as *Q*′. Calculate Δ*Q* = *Q*′−*Q*.

Step 2. Obtain items in List 2 of quantity Δ*Q* in order until the associated quantity of an item equals Δ*Q*. Form trajectories for the items in List 2. The last item is an end of the trajectory. If a single item is obtained, the associated trajectory ends.

Step 3. For items except the last item, let q be the associated quantity, calculate Δ*Q* = Δ*Q*−*q*, and repeat Step 2.

Step 4. Calculate the total cost for each trajectory in the obtained tree and select the minimum. The associated selections along the trajectory form the solution for a combination in Step 1.

Step 5. Compare the minimums in Step 4 for all combinations in Step 1 and obtain the minimum solution.

Step 2–4 in RGDQD are the same as step 2–4 in IGDQD that the operations on List 2 are identical. Step 1 enumerates different combinations in List 1. As *d*_1_ increases to *l*, total enumeration of *l*+*d*_2_ quantities is performed. As *d*_1_ and *d*_2_ decrease to 0, Step 1 becomes the Step 1 in IGDQD. The larger the parameters *d*_1_ and *d*_2_, the more combinations and the better the result. The extreme case is *d*_1_ = *l* and *d*_2_ = *N*−*l* so that all suppliers are considered. However, as *d*_1_ and *d*_2_ increase, the number of enumerated combinations also increases. The selection of *d*_1_ and *d*_2_ takes into account the average number of suppliers in an order for a particular *Q*, and the tradeoff between optimality and simplicity of the computations.

## Numerical study

A numerical example is presented for illustrative purposes. An order of 52 products arrives. After screening the technical requirements, 7 suppliers are available for selection. These suppliers are grouped by discount types, numbered 1 to 7, and denoted by S1 to S7. S1 offers no discount, S2, S3, and S4 offer the all-units discount, S5 and S6 with incremental discount, and S7 with carload discount. For simplicity and without loss of generality, all suppliers have a setup cost of $10 and a capacity of 20 units. Except for S1, the rest suppliers have 2 discount intervals. All boundary quantities of the first interval are 10 units. The unit costs are $2.3, $2.2, $1.9, $2.4, $2.1, $2.3, and $2.3 for S1 to S7. The discounted costs are $1.7, $1.8, $2.2, $1.9, $2, and $2.2 for S2 to S7. The actual unit cost is denoted by “auc”.

First, SGDQD is applied. List 1 is obtained: S2, 20 units, auc 2.2; S3, 20 units, auc 2.3. ΔQ = 52-20-20 = 12. One item list 2 of 12 is obtained: S7, 10 units, auc 2.84. ΔQ = 12–10 = 2. One item list 2 of 2 is obtained: S5, 2 units, auc 7.1. ΔQ = 2–2 = 0 and the algorithm terminates. The solution is: S2, 20 units; S3 20 units; S7 10 units; S5 2 units. The cost is $132.6. Then, IGDQD is applied. List 1 is the same. List 2 of 12 is obtained: S7, 10 units, auc 2.84; S5, 12 units, auc 2.9. Two trajectories are formed. The upper is the same as SGDQD. The costs of $132.6 and $124.8 are compared. The solution is S2, 20 units; S3 20 units; S5 12 units; cost $124.8. RGDQD is applied with ***d***_**1**_ = 1 and ***d***_**2**_ = 1. The enumerations are: S2, 20 units, S3, 20 units; S2, 20 units, S5, 20 units. The first combination is the same as in IGDQD. For the second combination, obtain List 2 of 12: S3, 12 units, auc 2.63. The cost is $125.6. The first combination gives lower cost and the solution is the same as IGDQD. The solutions by IGDQD and RGDQD are optimal.

## Tests instances

This section conducts computational experiments to show the performance of the proposed algorithms in solving the large-scale model (P).

### Random data generation

Supplier data are retrieved from the database and after supplier screening, 30% of suppliers offer no discount, 30% all-units discount, 30% incremental discount, and 10% carload discounts. The supplier data in the study are generated randomly as shown in Table 6.

**Table 6:** Random data generation.

Parameters	Random data generation
*S*_*i*_	[1000, 2000], uniform, integer
*c*_*i*_	[10, 20], uniform, one decimal point
Capacity	[400, 100000], uniform, multiples of 100
*M*_*i*_ carload discount	{3,5,7}, uniform
*M*_*i*_ other discounts	[2, 4], uniform, integer
*c*_*i*1_	[10.5, 20.5], uniform, one decimal point
*c*_*ij*_, *j*>1	*αc*_*i*1_
*b*_*ij*_ carload discount, *j* odd, *j*<*M*_*i*_	*b*_*ij*_ = 0.95×*b*_*i*,*j*+1_
*b*_*ij*_ others	Capacity uniformly divided to *M*_*i*_ intervals, rounded to multiples of 100

In Table 6, the discount rate α is generated as follows. The range of discount rate is [0.88, 0.99]. This range is divided by *M*_*i*_−1. Uniform dividing points are obtained which are rounded to two decimal points, e.g., for *M*_*i*_ = 4, the dividing points are 0.92 and 0.95. α values are randomly generated uniformly between the dividing points, e.g., for *M*_*i*_ = 4, discount rates α’s are generated uniformly between [0.95,0.99], [0.92,0.95], and [0.88,0.92].

### Solution quality of the proposed algorithms

20 instances of data of 1000 suppliers are randomly generated. Using the randomly generated data, 120 problems are formulated with different *Q*’s, i.e., *Q* = 3000, 15000, 75000, 150000, 300000, 600000. SGDQD, IGDQD, and RGDQD are applied to solve the 120 problems. RGDQD is applied with parameters *d*_1_ set to 1 and *d*_2_ set to 2.

The solutions are compared with the optimal solutions to evaluate the solution quality of the algorithms. Tables 7–12 give the solutions. The optimal solutions are in bold.

**Table 7:** Solutions for Q = 3000.

Data No.	Optimal	SGDQD	IGDQD	RGDQD
1	**31238**	**31238**	**31238**	**31238**
2	**31006**	**31006**	**31006**	**31006**
3	**31335**	**31335**	**31335**	**31335**
4	**31382**	**31382**	**31382**	**31382**
5	**31057**	**31057**	**31057**	**31057**
6	**31076**	**31076**	**31076**	**31076**
7	**29795**	**29795**	**29795**	**29795**
8	**31607**	**31607**	**31607**	**31607**
9	**31629**	**31629**	**31629**	**31629**
10	**30942**	**30942**	**30942**	**30942**
11	**31400**	**31400**	**31400**	**31400**
12	**31762**	**31762**	**31762**	**31762**
13	**30071**	**30071**	**30071**	**30071**
14	**29081**	**29081**	**29081**	**29081**
15	**31403**	**31403**	**31403**	**31403**
16	**31341**	**31341**	**31341**	**31341**
17	**31109**	**31109**	**31109**	**31109**
18	**31389**	**31389**	**31389**	**31389**
19	**31179**	**31179**	**31179**	**31179**
20	**30681**	**30681**	**30681**	**30681**

**Table 8:** Solutions for Q = 15000.

Data No.	Optimal	SGDQD	IGDQD	RGDQD
1	**145822**	**145822**	**145822**	**145822**
2	**146137**	146189	**146137**	**146137**
3	**145898**	**145898**	**145898**	**145898**
4	**144982**	145891	**144982**	**144982**
5	**148343.5**	148961.5	**148343.5**	**148343.5**
6	**140676**	**140676**	**140676**	**140676**
7	**147085.5**	149087	**147085.5**	**147085.5**
8	**144191**	**144191**	**144191**	**144191**
9	**147238**	147295	147295	147295
10	**145206**	**145206**	**145206**	**145206**
11	**145103**	145872	**145103**	**145103**
12	**142351**	**142351**	**142351**	**142351**
13	**147293**	**147293**	**147293**	**147293**
14	**143725**	144167	**143725**	**143725**
15	**148883**	**148883**	**148883**	**148883**
16	**142002**	**142002**	**142002**	**142002**
17	**144325**	**144325**	**144325**	**144325**
18	**142749**	**142749**	**142749**	**142749**
19	**146674**	147967	**146674**	**146674**
20	**144320**	**144320**	**144320**	**144320**

**Table 9:** Solutions for Q = 75000.

Data No.	Optimal	SGDQD	IGDQD	RGDQD
1	**706397**	**706397**	**706397**	**706397**
2	**691434**	**691434**	**691434**	**691434**
3	**691254**	**691254**	**691254**	**691254**
4	**698860**	701363	**698860**	**698860**
5	**697516**	**697516**	**697516**	**697516**
6	**698667**	**698667**	**698667**	**698667**
7	**703511**	705159.5	704614	704614
8	**700558**	706687.5	705160.5	**700558**
9	**701715**	705816	705816	705816
10	**710239**	**710239**	**710239**	**710239**
11	**702972**	705504	705504	704481
12	**699236**	**699236**	**699236**	**699236**
13	**707167**	**707167**	**707167**	**707167**
14	**718906**	722136	722136	722136
15	**691061**	**691061**	**691061**	**691061**
16	**702580**	**702580**	**702580**	**702580**
17	**691483**	**691483**	**691483**	**691483**
18	**706281**	**706281**	**706281**	**706281**
19	**693744**	**693744**	**693744**	**693744**
20	**704195**	**704195**	**704195**	**704195**

**Table 10:** Solutions for Q = 150000.

Data No.	Optimal	SGDQD	IGDQD	RGDQD
1	**1424483**	**1424483**	**1424483**	**1424483**
2	**1389968**	**1389968**	**1389968**	**1389968**
3	**1390136**	1405015	1402977	1391856
4	**1399213**	1399973	1399973	**1399213**
5	**1409111**	**1409111**	**1409111**	**1409111**
6	**1399675**	1403429	1402678	1402678
7	**1413685**	1422502	1422502	1418933
8	**1396483**	1406233	1404386	**1396483**
9	**1406630**	1409487	1409487	1409487
10	**1426813**	**1426813**	**1426813**	**1426813**
11	**1408390**	1409603	1409603	**1408390**
12	**1405337**	**1405337**	**1405337**	**1405337**
13	**1419313**	**1419313**	**1419313**	**1419313**
14	**1439665**	1439749	1439749	**1439665**
15	**1400747**	**1400747**	**1400747**	**1400747**
16	**1414164**	**1414164**	**1414164**	**1414164**
17	**1385024**	1385897	1385897	**1385024**
18	**1419529**	1428687	1428687	1421099
19	**1392620**	**1392620**	**1392620**	**1392620**
20	**1417920**	**1417920**	**1417920**	**1417920**

**Table 11:** Solutions for Q = 300000.

Data No.	Optimal	SGDQD	IGDQD	RGDQD
1	**2867289**	2870535	2870535	2867337
2	**2818138**	2821720	**2818138**	**2818138**
3	**2815867**	**2815867**	**2815867**	**2815867**
4	**2819017**	**2819017**	**2819017**	**2819017**
5	**2846407**	**2846407**	**2846407**	**2846407**
6	**2823178**	2826459	2826459	**2823178**
7	**2842147**	**2842147**	**2842147**	**2842147**
8	**2802754**	**2802754**	**2802754**	**2802754**
9	**2835426**	**2835426**	**2835426**	**2835426**
10	**2892312**	2894610	**2892312**	**2892312**
11	**2830856**	2836030	**2830856**	**2830856**
12	**2832963**	**2832963**	**2832963**	**2832963**
13	**2859533**	2862234	2862234	2859589
14	**2895243**	2896346	**2895243**	**2895243**
15	**2832040**	**2832040**	**2832040**	**2832040**
16	**2876920**	2877209	2877055	2877055
17	**2836755**	2838494	**2836755**	**2836755**
18	**2859071**	2859683	2859683	2859683
19	**2795631**	**2795631**	**2795631**	**2795631**
20	**2864130**	2867909	2867909	2867909

**Table 12:** Solutions for Q = 600000.

Data No.	Optimal	SGDQD	IGDQD	RGDQD
1	**5793760**	5795388	5795388	**5793760**
2	**5722664**	**5722664**	**5722664**	**5722664**
3	**5711887**	**5711887**	**5711887**	**5711887**
4	**5712787**	5712895	5712895	5712895
5	**5771409**	5775961	5775961	**5771409**
6	**5711574**	5712156	5712156	5712156
7	**5752831**	5758116	5757365	5752969
8	**5670679**	**5670679**	**5670679**	**5670679**
9	**5778432**	**5778432**	**5778432**	**5778432**
10	**5853339**	5855204	5854472	**5853339**
11	**5730216**	5733588	5733588	5730882
12	**5779731**	5782190	5781741	5781741
13	**5778738**	**5778738**	**5778738**	**5778738**
14	**5862801**	5864551	5864551	5864551
15	**5768889**	5771004	5771004	5771004
16	**5842383**	5844585	5844580	5844580
17	**5822320**	5823688	5823688	**5822320**
18	**5769214**	**5769214**	**5769214**	**5769214**
19	**5648811**	5649294	5649294	5649294
20	**5793635**	5798220	5793777	**5793635**

The optimality gap is calculated as follows,

$$Opt.Gap=\frac{\left|solution-Optimal\right|}{Optimal}.$$

**Fig 7:**
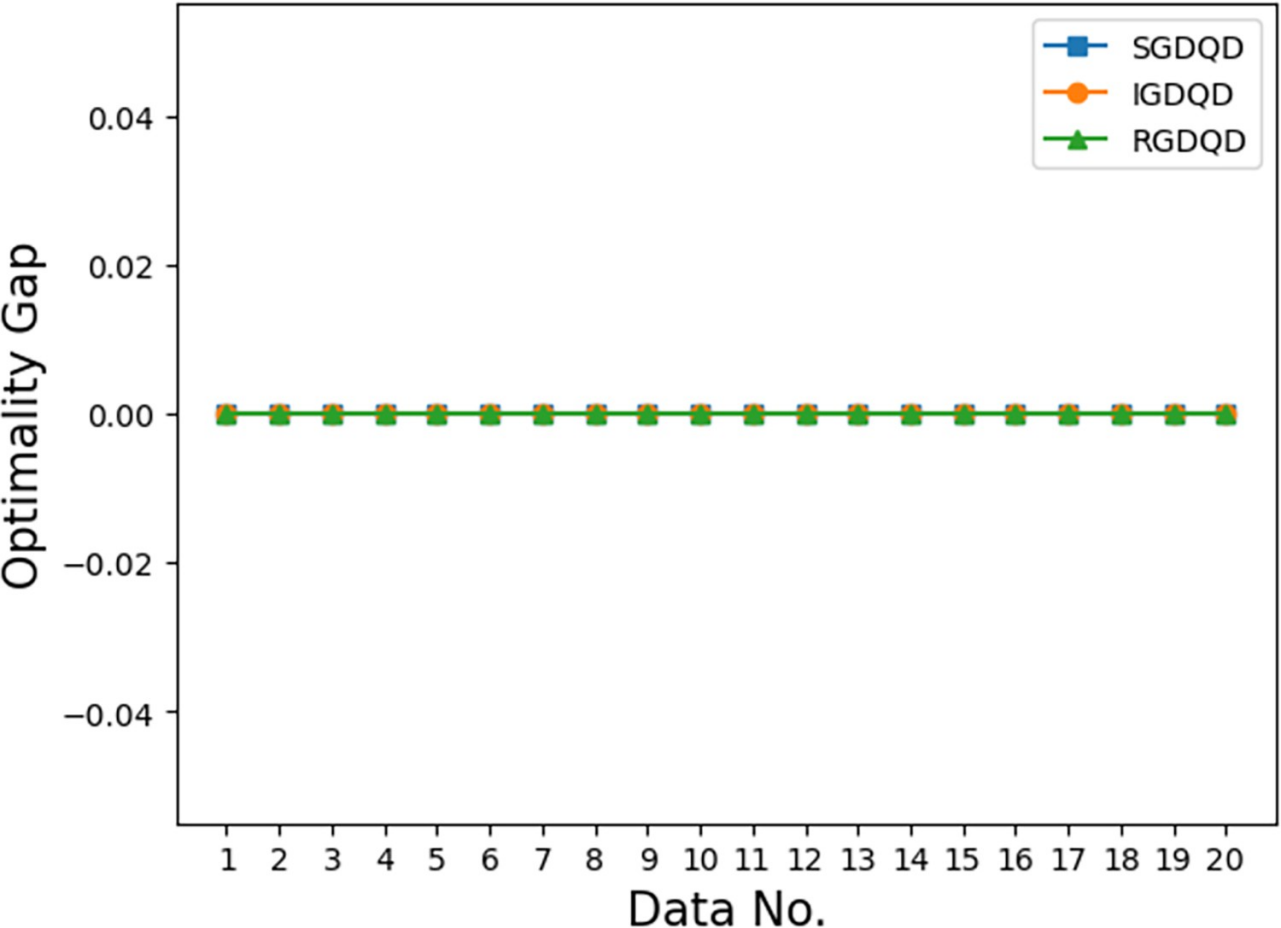
Comparison of optimality gaps for *Q* = 3000.

**Fig 8:**
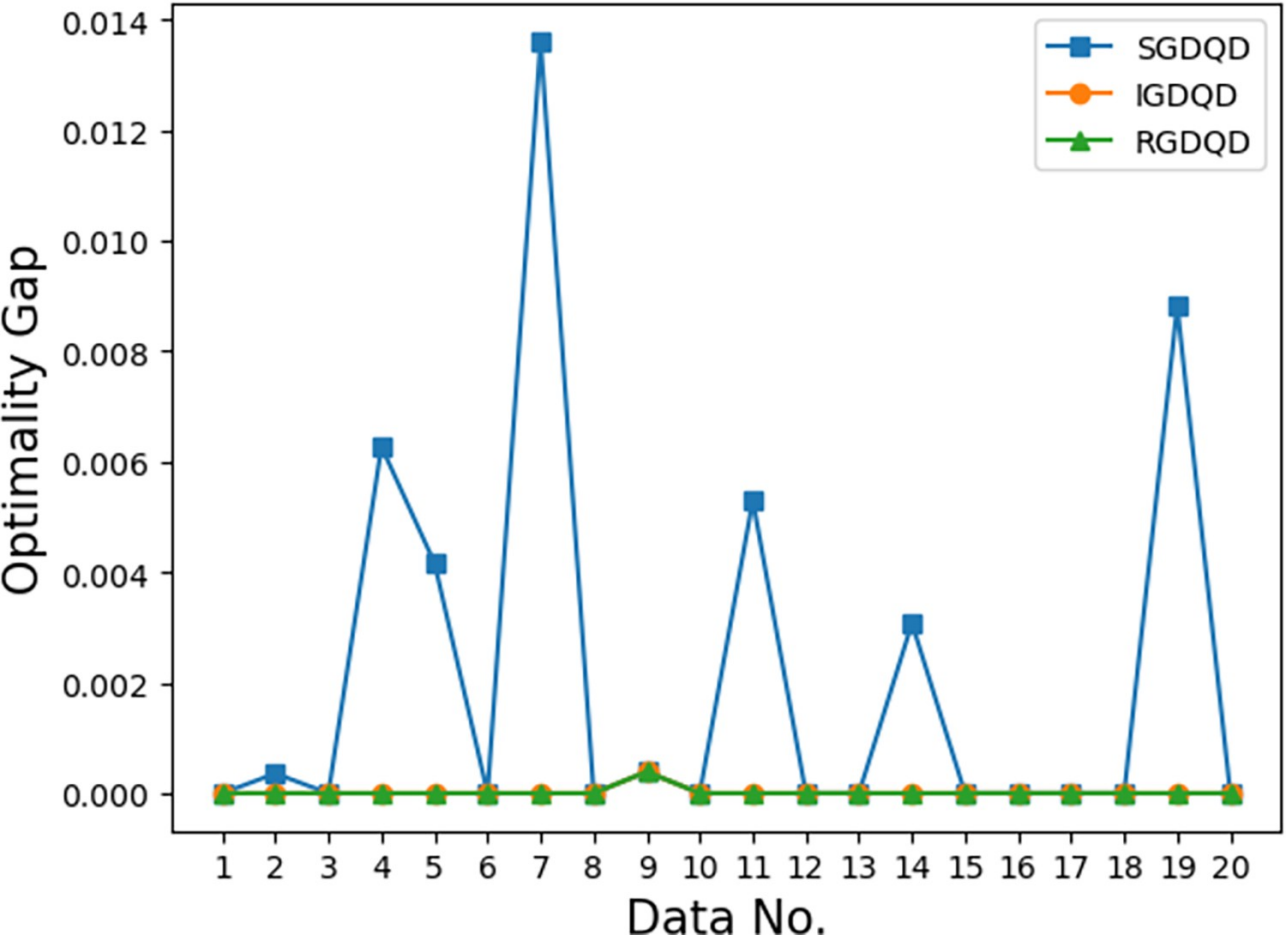
Comparison of optimality gaps for *Q* = 15000.

**Fig 9:**
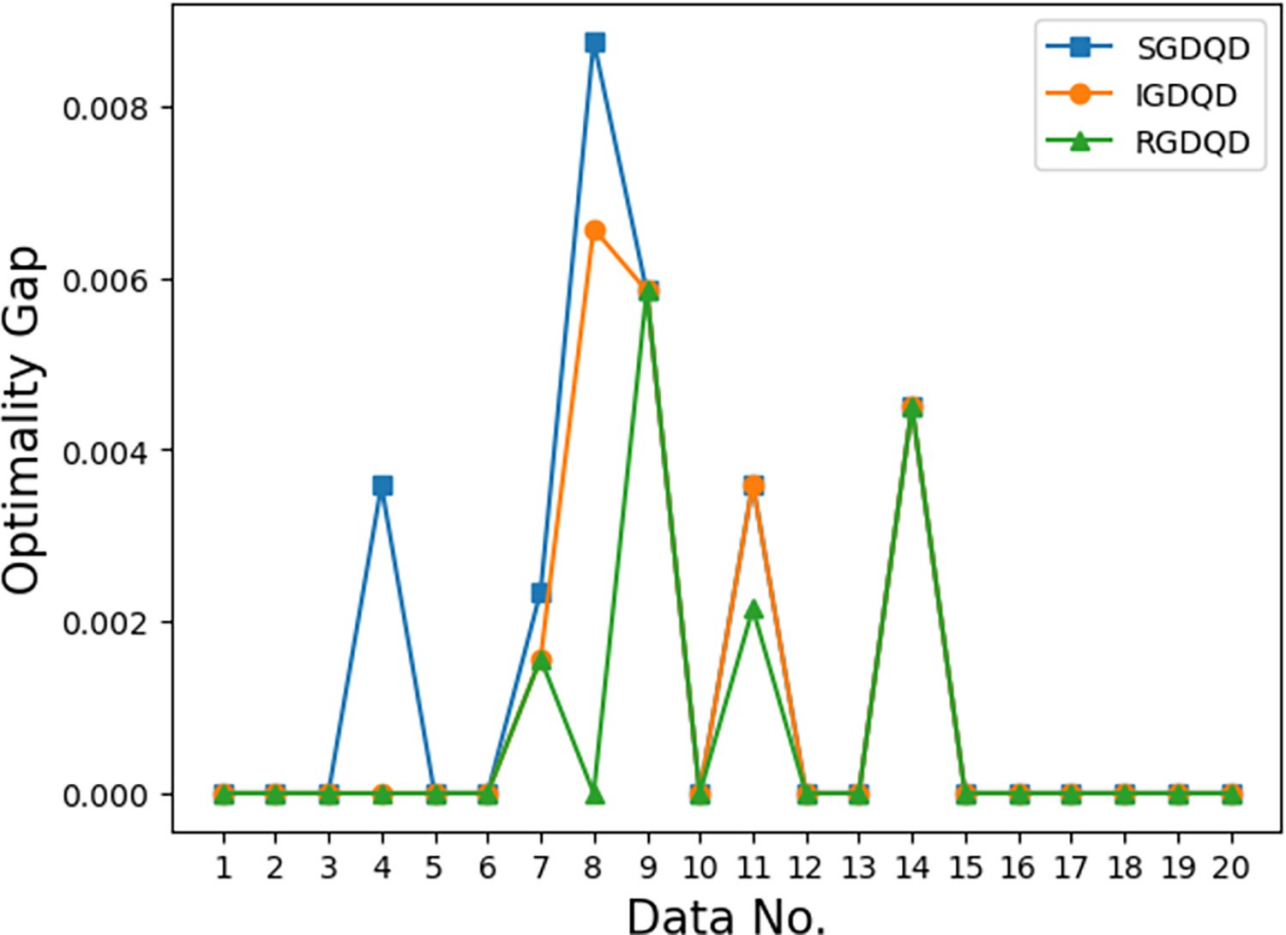
Comparison of optimality gaps for *Q* = 75000.

**Fig 10:**
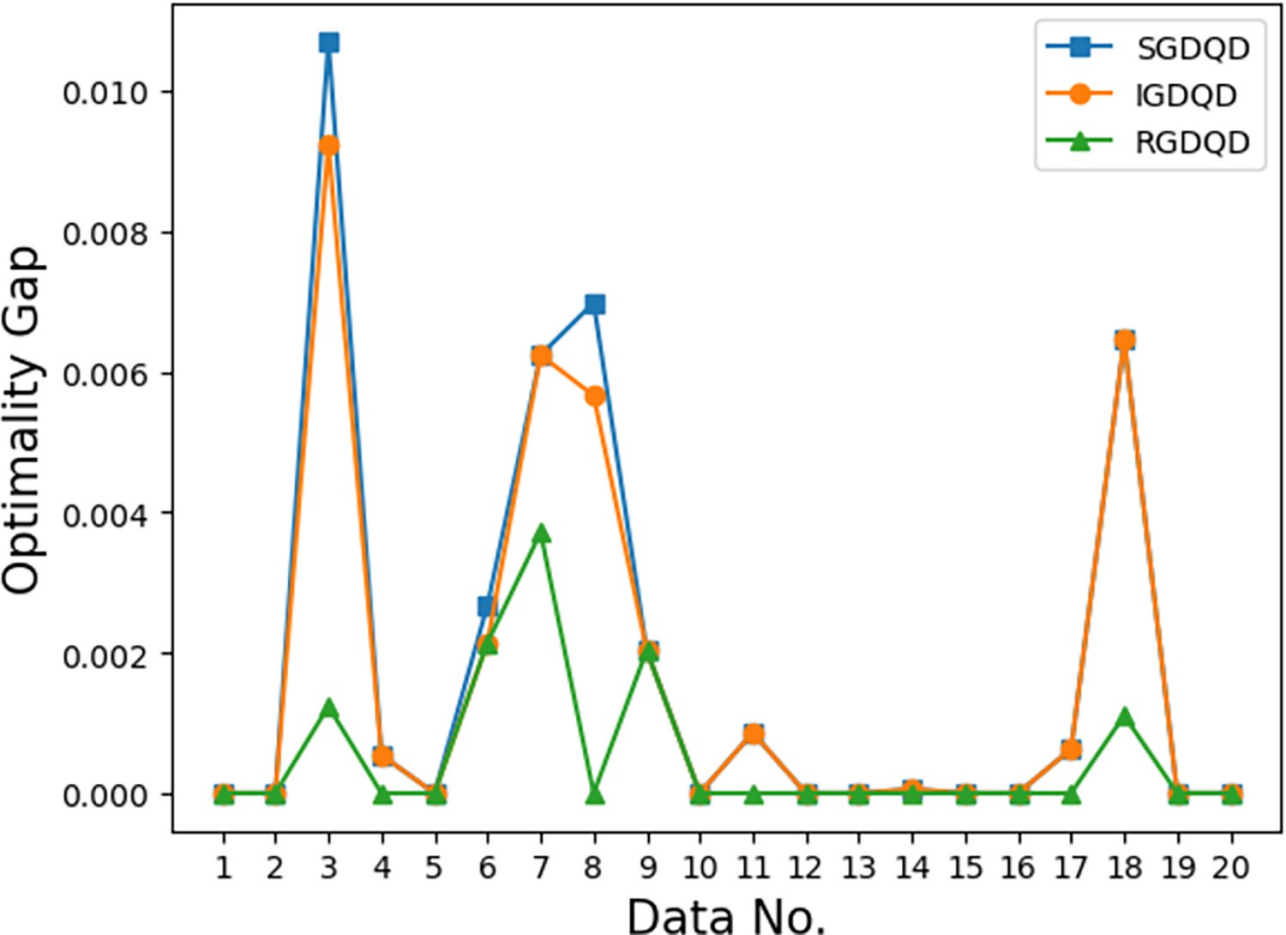
Comparison of optimality gaps for *Q* = 150000.

**Fig 11:**
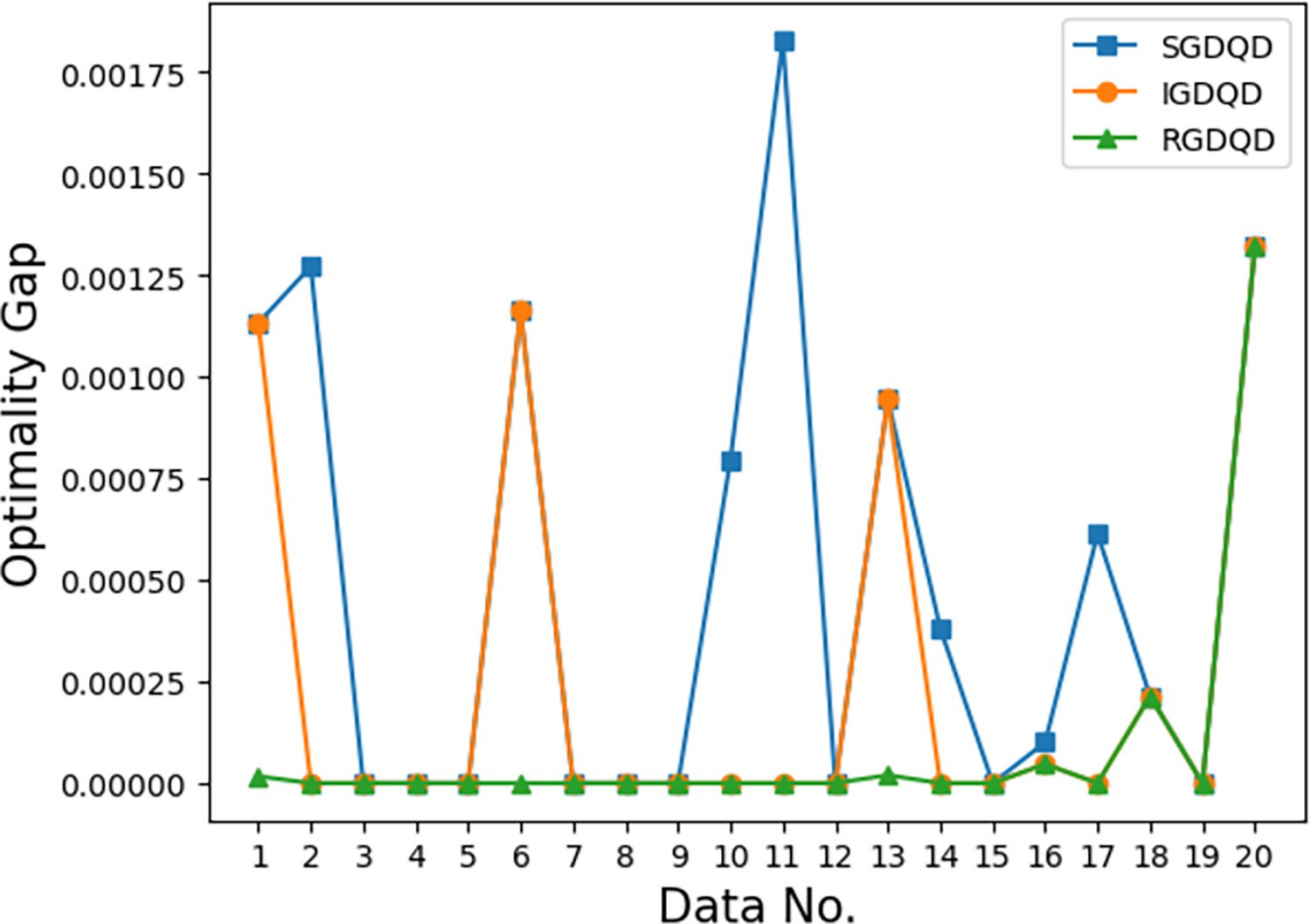
Comparison of optimality gaps for *Q* = 300000.

**Fig 12:**
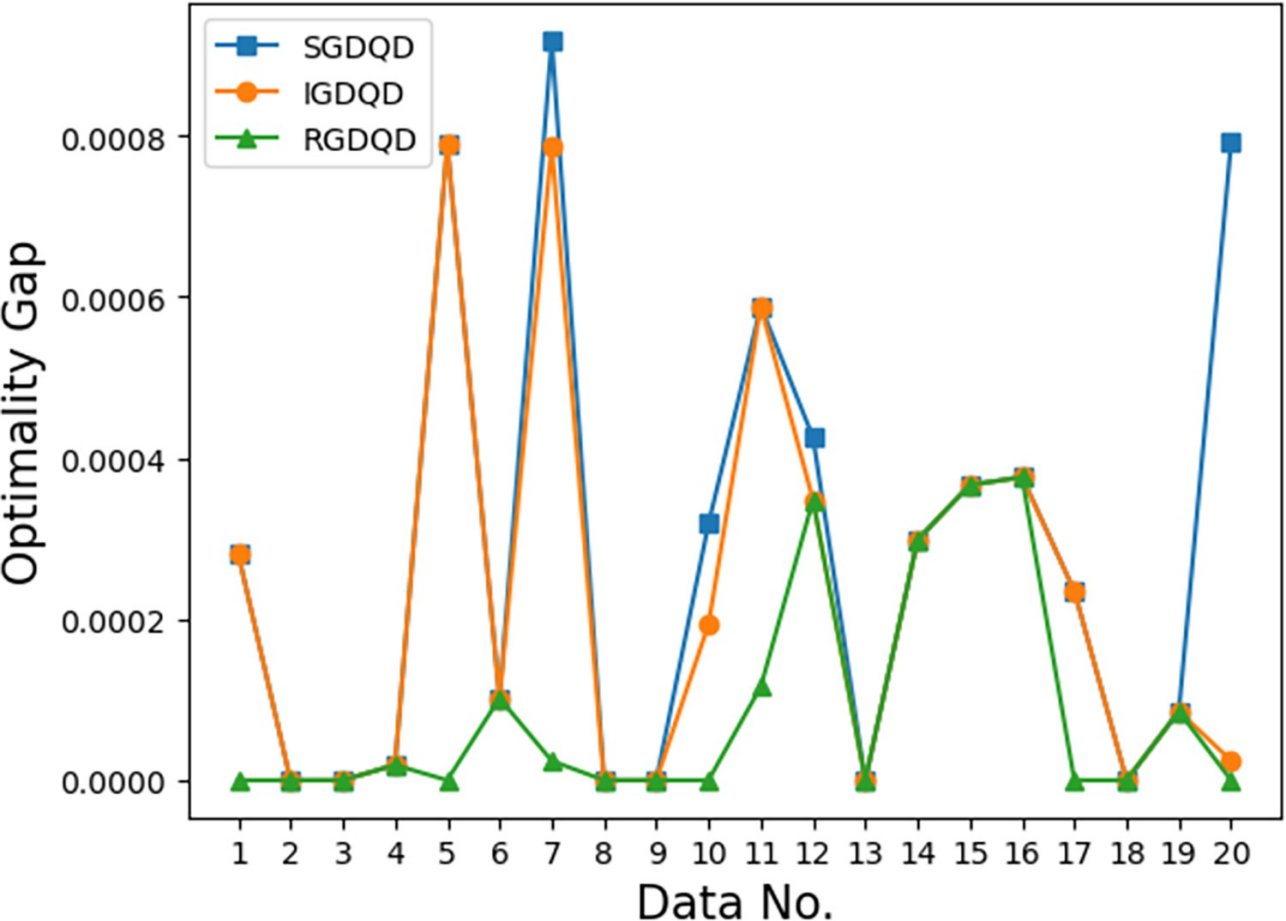
Comparison of optimality gaps for *Q* = 600000.

Figs 7–12 show the improvements in solution quality with narrowing optimality gaps solved by SGDQD, IGDQD, and RGDQD. Tables 13–15 show the relevant statistics.

**Table 13:** Statistics of optimality gaps for SGDQD.

*Q*	Average	s.d.	Average (Nonzero)	s.d. (Nonzero)	Largest	Smallest	Percentage of Opt. Solutions
3000	0	0	/	/	0	0	100%
15000	0.002099	0.003765	0.005247	0.004426	0.013608	0	60%
75000	0.001431	0.002526	0.004769	0.002269	0.008749	0	70%
150000	0.001859	0.003141	0.003718	0.003626	0.010703	0	50%
300000	0.000488	0.000592	0.000887	0.000526	0.001828	0	45%
600000	0.000280	0.000297	0.000400	0.000278	0.000919	0	30%
overall	0.001026	0.002363	0.002513	0.002541	0.013608	0	59.2%

**Table 14:** Statistics of optimality gaps for IGDQD.

*Q*	Average	s.d.	Average (Nonzero)	s.d. (Nonzero)	Largest	Smallest	Percentage of Opt. Solutions
3000	0	0	/	/	0	0	100%
15000	0.000019	0.000087	0.000387	/	0.000387	0	95%
75000	0.001104	0.002159	0.004415	0.001966	0.006570	0	75%
150000	0.001693	0.002817	0.003385	0.003222	0.009237	0	50%
300000	0.000241	0.000467	0.000803	0.000537	0.001319	0	70%
600000	0.000225	0.000257	0.000321	0.000252	0.000789	0	30%
overall	0.000547	0.001569	0.001823	0.002444	0.009237	0	70%

**Table 15:** Statistics of optimality gaps for RGDQD.

*Q*	Average	s.d.	Average (Nonzero)	s.d. (Nonzero)	Largest	Smallest	Percentage of Opt. Solutions
3000	0	0	/	/	0	0	100%
15000	0.000019	0.000087	0.000387	/	0.000387	0	95%
75000	0.000703	0.001647	0.003513	0.002004	0.005844	0	80%
150000	0.000512	0.001027	0.002046	0.001040	0.003712	0	75%
300000	0.000081	0.000295	0.000323	0.000563	0.001319	0	75%
600000	0.000087	0.000139	0.000193	0.000151	0.000376	0	55%
overall	0.000234	0.000833	0.001168	0.001566	0.005844	0	80%

Tables 7–12 and Figs 7–12 show that the solutions obtained by the proposed greedy algorithms are optimal or very close to optimal. In the statistics of Tables 13–15, 59.2%, 70%, and 80% of the solutions are optimal solutions solved by SGDQD, IGDQD, and RGDQD respectively. The average optimality gaps are only 0.001026, 0.000547, and 0.000234 for SGDQD, IGDQD, and RGDQD respectively, with standard deviations of 0.002363, 0.001569, and 0.000833 respectively. The solution quality improves from SGDQD to IGDQD and from IGDQD to RGDQD.

In the simulations, *d*_1_ and *d*_2_ in RGDQD are set to small values, 1 and 2. Results show that by simple enumeration, where both *d*_1_ and *d*_2_ bear small values, improvements in solution quality are obvious. Increasing these values gives better results. For instance, if *d*_1_ is set to 7 and *d*_2_ is set to 6, 90% of the solutions will be optimal.

#### Further improvements

Further improvements of RGDQD are possible through some more observations. For instance, it is often observed that for the carload discount, although an even interval may have a smaller actual unit cost than the next odd interval, the quantity of the odd interval constitutes a larger proportion of *Q*. Choosing it often yields better results. Thus, the following step may be added to Step 1 of RGDQD:

Step 1^+^. If both an even interval and its next odd interval of carload discount are present above the order *l*+*d*_2_ in List 1, and the even interval is above the odd interval, then the even interval can be replaced by its next odd interval and obtain the combinations.

**Fig 13:**
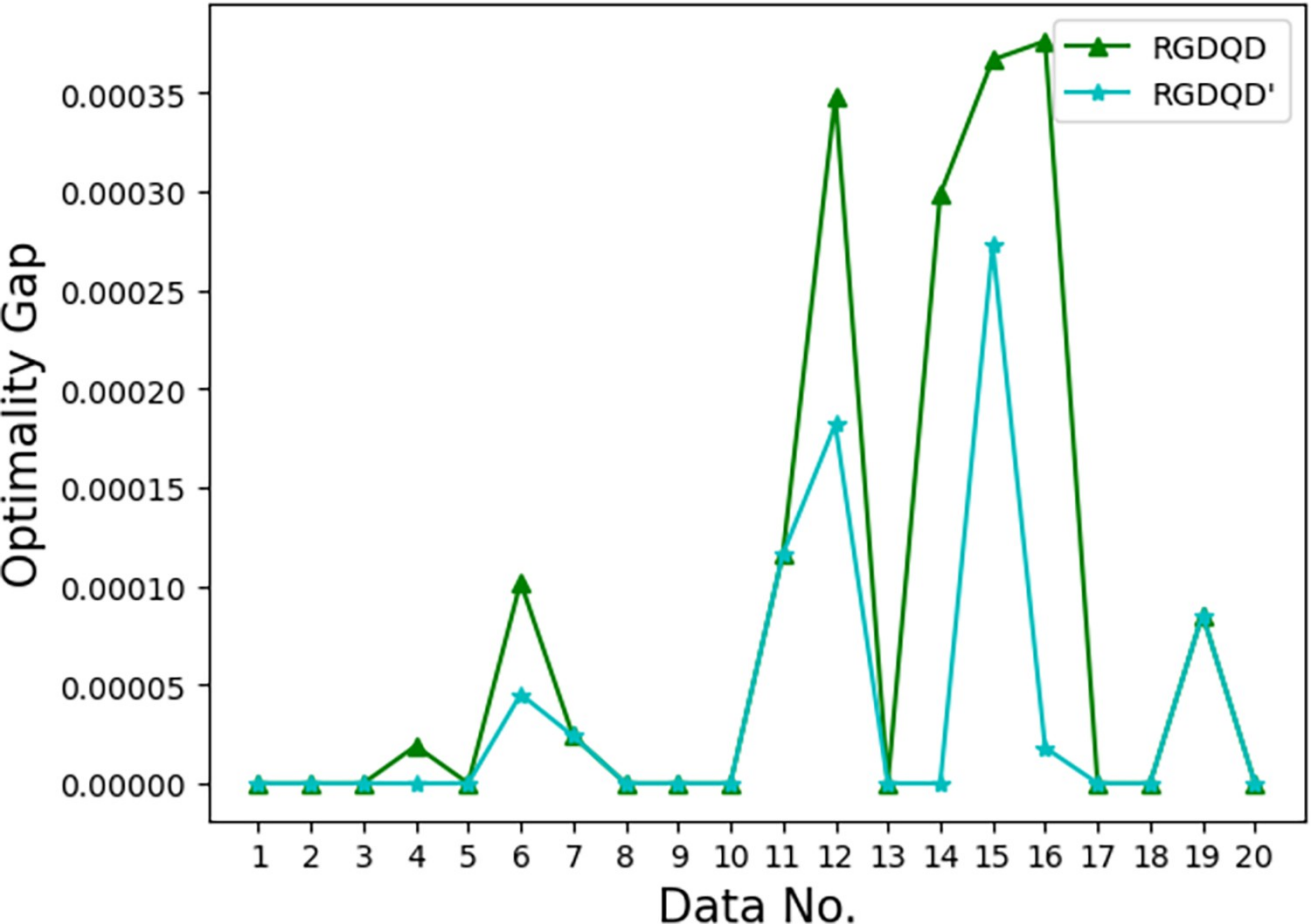
Comparison of optimality gaps for RGDQD and RGDQD’.

The effect of adding Step 1^+^ to RGDQD is tested for *Q* = 600000. The resulting algorithm is termed RGDQD’. Fig 13 shows the improvements in solution quality solved by RGDQD’ compared with the original RGDQD.

### The efficiency of the proposed algorithms

To test the efficiency of the proposed algorithms, the number of suppliers *N* is set to 1000, 10000, and 100000. 10 instances of each are generated. The order quantity *Q* is set to 10000, 100000, and 1000000. The algorithms are coded in Python and the simulation is conducted on a personal computer, Intel core i5-7200U, 2.5GHz 2.71GHz, 8GB RAM. The solution time is the average of 10 runs with generated instances for each combination of the two parameters. The average solution time of the three proposed algorithms is listed in Table 16.

**Table 16:** Average solution time of the proposed algorithms (seconds).

	SGDQD	IGDQD	RGDQD
*N* = 1000, *Q =* 10000	0.03	0.03	0.03
*N* = 1000, *Q* = 100000	0.03	0.04	0.05
*N* = 1000, *Q* = 1000000	0.04	0.05	0.05
*N* = 10000, *Q =* 10000	0.33	0.34	0.36
*N* = 10000, *Q* = 100000	0.33	0.36	0.40
*N* = 10000, *Q* = 1000000	0.43	0.43	0.49
*N* = 100000, *Q =* 10000	3.20	3.25	3.32
*N* = 100000, *Q* = 100000	3.39	3.37	3.71
*N* = 100000, *Q* = 1000000	4.35	4.38	4.54

Table 16 shows that with *N* = 1000, (P) can be solved in centiseconds, and with *N* = 10000, (P) can be solved in less than half a second. Even with a very large dimension–up to 100000 suppliers, (P) can still be solved in several seconds. Table 16 also shows that the running time increases slowly with the increase of the order quantity *Q*.

### Comparison with the genetic algorithm

The genetic algorithm (GA) has been an excellent and widely applied solution mechanism for combinatorial optimization problems [14, 22, 23]. Compared to traditional algorithms, GA tends to solve problems more efficiently. Although GA provides a general evolution mechanism, the design of GA is problem specific. [14] applied GA to solve the proposed NP-hard mixed integer nonlinear programming model for the multiple sourcing problem with supplier failure risk and quantity discount. The problem in this study has only 10 suppliers. [34] designed a hybrid genetic algorithm to solve the two-dimensional single large object placement problem and showed its good performance in terms of solution time and quality compared to other algorithms. In [35], a variable-grouping based GA for large-scale integer programming was proposed that outperformed the standard GA. Quadratic knapsack problems with less than 400 variables were solved with a solution time of up to 9726 seconds.

To compare with the proposed greedy algorithms, attempts of applying GA to solve the large-scale (P) are made with Geatpy2—the genetic and evolutionary algorithm toolbox for Python with high performance. Geatpy2 is a GA toolbox that provides GA templates with adjustable operators and delivers leading solution performance. Details of the toolbox can be found at http://geatpy.com/ and resources can be found in the geatpy-dev/geatpy directory on GitHub. For all the trials, feasible solutions are not able to be found. Applying GA to solve large dimensional nonlinear integer programming problems remains a challenge. The proper design of GA to solve (P) becomes another interesting research topic.

The proposed greedy algorithms provide a very good solution substitute when other algorithms are unable to find optimal or even feasible solutions for the large-scale (P).

### Sensitivity analysis

Sensitivity analysis is performed to study the impact of parameters on solution quality and the final solution.

#### Sensitivity analysis of the order quantity *Q* on solution quality

A sensitivity analysis of *Q* on solution quality is performed. Holding other values constant, Tables 13–15 show that the optimality gaps generally decrease as *Q* increases. But the percentage of optimal solutions also decreases with increasing *Q*.

**Fig 14:**
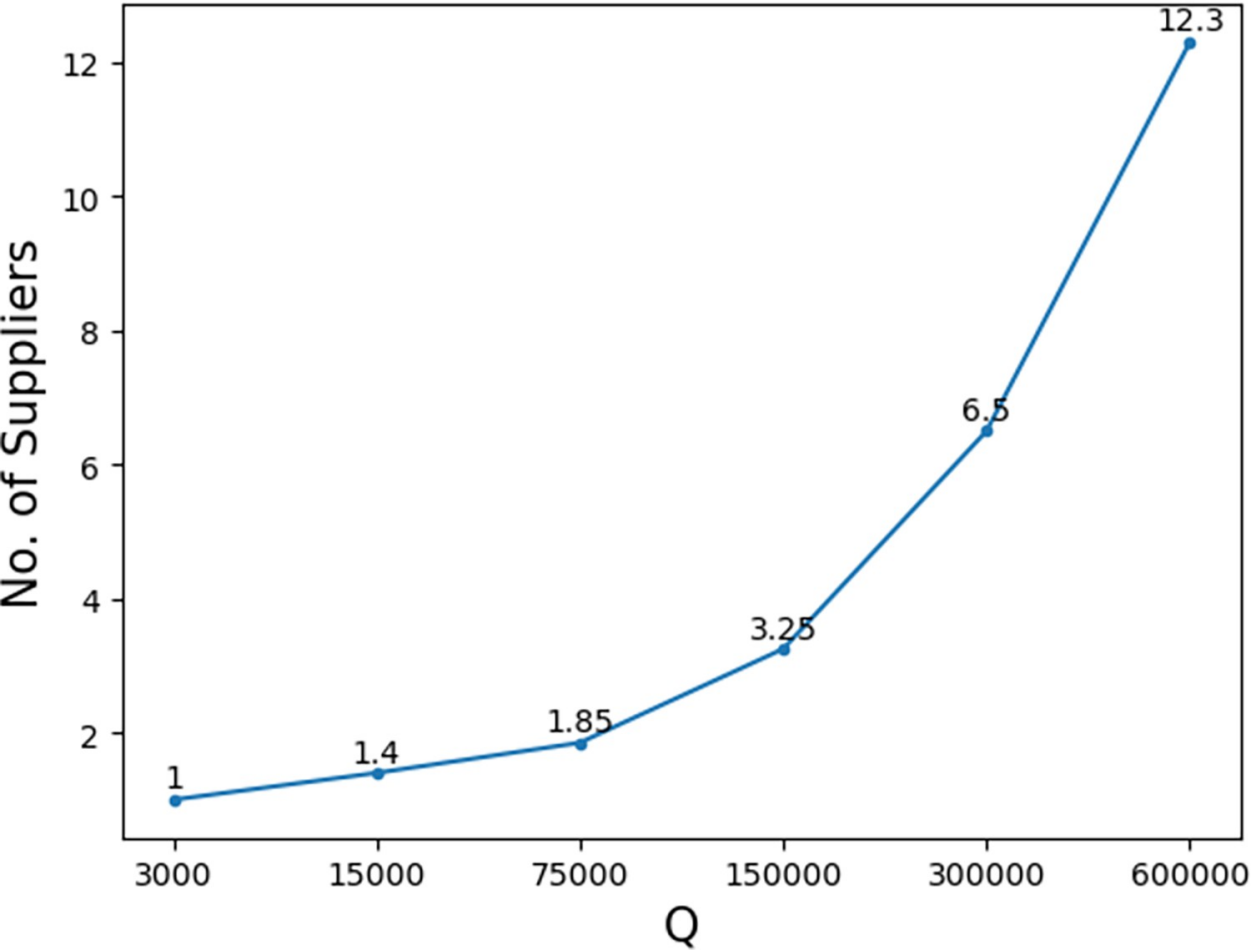
The average number of suppliers in the optimal solutions for different *Q*’s.

For very small *Q*, i.e., *Q* = 3000, all solutions are optimal. Small or large *Q* corresponds to whether the average number of suppliers in the optimal solution is small or large, refer to Fig 14. For *Q* = 3000, the optimal solution has only one supplier. (P) becomes a supplier selection problem with no order allocation. Table 13 shows that when SGDQD selects only one supplier, it obtains optimal solutions for all cases. The other two algorithms are improvements of SGDQD and thus both obtain optimal solutions.

As *Q* increases to 15000, it is still quite small and there are 1.4 suppliers in the optimal solution on average. Only 60% of the solutions achieve optimality for SGDQD. All the solutions are optimal if only one supplier is selected. If more suppliers are involved, all the solutions obtained are nonoptimal. The average optimality gap of these solutions is 0.5247%, compared to 0.4769%, 0.3718%, 0.0887%, and 0.0400% for other order quantities. With a small *Q*, the selection of non-optimal suppliers contributes larger to the solution. This can be tackled by IGDQD. 95% of the solutions are optimal for IGDQD and RGDQD. With more calculations performed, optimal solutions are obtained generally. The only nonoptimal solution has a small optimality gap.

With the further increase of *Q*, the solution quality generally improves. The trend becomes more pronounced when the order quantity becomes large. The reason is that as more suppliers join to fill *Q*, a larger percentage of suppliers are the same as in the optimal solutions. It results in smaller optimality gaps. The improvements by IGDQD and RGDQD are not as large for *Q* = 15000.

#### Sensitivity analysis of parameters on the final solution

By changing the parameters in the numerical example, the selected suppliers may change. Table 17 shows the change of parameters and the corresponding optimal solutions.

**Table 17:** Solutions of the numerical example by changing its parameters.

Change of parameter	Solution
*S*_6_ = 5	S2 20, S3 20, S6 12
*c*_42_ = 1.7	S2 20, S3 12, S4 20
*b*_22_ = 30	S2 30, S3 12, S7 10
*Q* = 67	S2 20, S3 20, S5 17, S7 10
*c*_21_ = 2.4, *c*_22_ = 2.3	S3 20, S4 12, S5 20

Analyzing the solutions of the computational tests, it is found that intervals with low unit costs are selected. Add the same amount to the randomly generated setup costs *S*_*i*_, and keep the other data constant, the selected suppliers’ intervals change for large amounts. It means that not only the unit costs take effect. By varying the scale of the other parameters, solution analyses show that actual unit costs are most important in the selection. The capacity of the supplier, the number of discount intervals, the discount rate, the setup cost alone, or the unit cost alone is not.

### Managerial insights

The results lead to the following managerial insights.

First, from the results in the “Tests instances” section, the proposed algorithms can assist managers in making timely decisions in the face of a huge supplier set. Supplier big data can be fully utilized. For very large-scale model solving, efficient algorithms are demanded.

Second, flexible supplier selection decisions are necessary to obtain the lowest cost allocation. The supplier screening process obtains different suppliers. The selected suppliers change with model parameter changes. In the big data era, where data are dynamically recorded, fixing the set of suppliers may not be a good choice.

Third, the order quantity affects the solution quality of the algorithms. When the average number of suppliers in the order are 1, the solutions by SGDQD are generally optimal. When the number is between 1 and 2, the solutions by IGDQD are generally optimal. If the order quantity is very small, SGDQD is the suitable algorithm with the greatest efficiency. If the quantity is small, IGDQD may be the choice. RGDQD gives the best solution quality. If the company receives orders with large amounts, RGDQD can be chosen for selection.

Last, using the actual unit costs to make greedy selections, the average optimality gaps of proposed greedy algorithms are only 0.1026%, 0.0547%, and 0.0234%. Moreover, sensitivity analysis shows that the actual unit costs play a major role in selecting the optimal suppliers. Therefore, if the company purchases internationally, which implies high setup costs (including transportation costs), the setup costs become more important in selecting suppliers. It is essential to consider not only the unit costs but also the suppliers’ distance and mode of transportation. If the suppliers are near the location of the company and setup costs are low, the company may select suppliers with low unit costs.

## Conclusion

In this paper, greedy algorithms are proposed to solve the formulated large-scale model with four quantity discount types for flexible supplier selection and order allocation in the big data era. Three greedy algorithms—SGDQD, IGDQD, and RGDQD are developed incrementally from the previous algorithm, utilizing more information and obtaining better performance. RGDQD is not yet the optimal algorithm, though it approaches optimality by increasing its parameters *d*_1_ and *d*_2_. The run time of the algorithm increases with the increase of the parameters. *d*_1_ and *d*_2_ in the study are set to small values– 1 and 2. For future research, the tradeoff between optimality and run time may be studied to find the best *d*_1_, *d*_2_ assignment for real applications. Existing models in the literature may be reformulated to take into account more quantity discounts. Efficient methodologies for solving large-scale models should be investigated in the era of big data.
